# Chronic and rare disease patients' access to healthcare services during a health crisis: The example of the COVID‐19 pandemic in Turkey

**DOI:** 10.1111/hex.13321

**Published:** 2021-07-26

**Authors:** Puren Aktas

**Affiliations:** ^1^ Department of Politics, School of Social Sciences The University of Manchester Manchester UK

**Keywords:** COVID‐19, healthcare access, patient experiences, patient organisations, qualitative, Turkey

## Abstract

**Objective:**

The restructuring of healthcare provision for the coronavirus disease 2019 (COVID‐19) pandemic caused disruptions in access for patients with chronic or rare diseases. This study explores the experiences of patients with chronic or rare diseases in access to healthcare services in Turkey during the COVID‐19 pandemic.

**Methods:**

Semi‐structured interviews were conducted with representatives (*n* = 10) of patient organisations (*n* = 9) based in Istanbul. Thematic analysis with an inductive approach was conducted to analyse the responses obtained through the interviews.

**Results:**

The lack of clinical information at the beginning of the pandemic caused fear among patients with chronic or rare diseases. Patients experienced obstacles in access to healthcare services because of the overcrowding of hospitals with COVID‐19 patients. Some treatment procedures were cancelled or postponed by physicians. Of these procedures, some were medically vital for those patients, leading to or exacerbating further health problems. The most positive measures that patients identified were where the Social Security Institution introduced regulations to facilitate access to prescribed medicine for chronic patients. Information exchange between the doctors and their patients was important to alleviate the uncertainty and reduce the anxiety among patients.

**Discussion:**

Access problems experienced by patients during the COVID‐19 pandemic were a complex mix of factors including shortages and physical barriers, but also perceptions of barriers. The findings of this study show that patient organisations can provide insights on disease‐specific experiences and problems that are very valuable to improve access to healthcare services to achieve the universal health coverage target. Hence, this study emphasises the inclusion of patient organisations in decision‐making processes during times of health crises.

**Public Contribution:**

Representatives of patient organisations participated in the interviews.

## 1 INTRODUCTION

The coronavirus disease 2019 (COVID‐19) pandemic has introduced challenges for all dimensions of healthcare systems, forcing countries to restructure the provision of services to meet urgent demands for preventing the spread of the virus and treating infected individuals. Hospitals were transformed into pandemic‐oriented hospitals, elective surgeries were cancelled, or postponed and face‐to‐face consultations were moved to virtual platforms. Many health systems experienced shortages of medical supplies, most importantly, intensive care unit (ICU) beds and ventilators, causing ethical dilemmas for health workers such as rationing of limited healthcare resources.^1^, ^2^

The shift in resources among healthcare systems has affected the delivery of clinical services to patients who did not have COVID‐19.^3^ It created disruptions in the continuum of care and delays in diagnosis procedures. Among patients who do not have COVID‐19, those with chronic diseases and rare diseases are the most vulnerable because of their complex health conditions and routine need to access specialised medical services.^4^, ^5^ Besides, patients with rare diseases need regular, multidisciplinary consultations conducted by a board of specialists and complex treatment services. Even during the regular functioning of healthcare systems, patients with rare diseases face significant challenges in access to healthcare services because of their complex healthcare situation, which requires multidisciplinary consultations, extensive screening and monitoring procedures and expensive treatments.^6^

During the pandemic, World Health Organization (WHO) suggested that countries identify context‐relevant essential services to prioritise for continuation, which includes the provision of medications, supplies and support from healthcare workers for the ongoing management of chronic diseases.^7^, ^8^ Identifying the issues that patients with chronic diseases might face, WHO listed some modifications to maintain essential services, which are better information provision to the patients about COVID‐19 and their disease‐specific conditions, raising awareness about telehealth or online services for regular monitoring or urgent care for acute exacerbations or deterioration, creation of self‐management and monitoring plans of the disease, increasing home supplies of medication and stocks of monitoring devices and modification of routine consultations.^8^

The problems that faced in response to the pandemic have been exacerbated by the neoliberal policies implemented in Western countries since the late 1970s.^9^ Privatisation of welfare services, cuts in public healthcare spending and divergence from the public health centralised approach resulted in a reduced ability to respond effectively to the pandemic.^10^ To respond to the pandemic's challenges, Navarro^9^ suggests the provision of universal health coverage (UHC) alongside other publicly provided welfare services. The UHC, by definition, indicates an ideal that ‘all people have access to the health services they need, when and where they need them, without financial hardship’.^11^ However, this aspirational definition overlooks an unexpected crisis, such as the COVID‐19 pandemic. The pandemic has introduced complex challenges to healthcare systems, interrupting citizens' access to healthcare services even in countries with UHC. These challenges give rise to the question of whether it is possible to ensure every citizen's access to healthcare services during an acute pandemic response considering the different needs and priorities coexisting within the same healthcare system under resource constraints.

The restructuring of healthcare services involves potential trade‐offs between ensuring access to healthcare services for every citizen and meeting the pandemic's requirements by shifting the provision of expensive and time‐consuming resources such as ICUs. This study explores patient experiences during the COVID‐19 pandemic in Turkey, with a focus on patients with chronic or rare diseases, considering their complex healthcare needs, which require specialist services. The findings derive from data collected through nine semi‐structured interviews conducted with 10 participants from patient organisations (POs) based in Istanbul. Drawing upon studies of the impact of the pandemic on patients with chronic or rare diseases, this article aims to contribute to the literature discussing the capacity of Turkey's healthcare system to meet the needs of citizens with complex healthcare needs as a country that provides UHC.

## 2 LITERATURE REVIEW

Concerns about the access of patients without COVID‐19 to healthcare services sparked a new corpus of research in medicine to explore the challenges faced by patients with chronic diseases. According to these studies, the pandemic caused obstacles in access to essential health services because of the shift of resource allocation to COVID‐19 services, limits on access to essential and nonessential services and cancellation or postponement of elective surgeries.^5^, ^12^, ^13^, ^14^, ^15^, ^16^ In a study conducted by Halley et al.,^12^ some patients and their relatives stated problems in access to essential medical supplies because of shortages. The inability to access their doctors not only worsened their health condition but also led to a sense of feeling neglected by healthcare providers.^12^ These issues have negative impacts on patients' health status,^12^, ^13^ which is also recognised by healthcare professionals.^14^ Considering their existing comorbidities, access problems might create life‐threatening challenges for patients with chronic or rare diseases.

Access problems not only affect patients in need of medical care but also those seeking a diagnosis or considering undergoing a diagnostic procedure for potential health problems.^12^, ^15^, ^17^, ^18^ Wingrove et al.^18^ surveyed organisations under the World Hepatitis Alliance to explore the impacts of the pandemic on viral hepatitis services and people living with viral hepatitis across the world. The results reveal problems in access to testing and to medication because of the closure of testing facilities, and lack of adequate information to individuals living with viral hepatitis. Delays in diagnosis cause concerns among health professionals because of potential increases in mortality from delayed treatment.^19^

Individuals with chronic and rare diseases already experience uncertainties about their health and future, which have been aggravated by the pandemic, such as the risk of contracting the infection, not being able to receive the needed care and lack of both adequate and conflicting information.^20^ These uncertainties, combined with social isolation, created new mental health challenges or worsened existing ones, as demonstrated by previous research.^13^, ^14^, ^21^, ^22^

In an effort to deal with problems in delivering face‐to‐face consultations, healthcare providers in many countries adopted virtual healthcare provision, known as telemedicine.^14^, ^23^ However, patients and their relatives are concerned about telemedicine as the primary method to access healthcare since they believe that it is insufficient for managing rare diseases considering the patients' complex healthcare conditions, which require monitoring and therapeutic services that cannot be easily transferred to online platforms.^12^ Additionally, virtualisation of the healthcare system exacerbates the risk of widening inequalities in access to healthcare, especially for individuals with worse health outcomes, considering the existing gaps in IT access between individuals with different levels of socioeconomic status.^24^

## 3 TURKISH HEALTHCARE SYSTEM AND ITS RESPONSE TO COVID‐19

Turkey, as an upper‐middle‐income country,^25^ introduced UHC in 2003 with a compulsory social health insurance scheme and equal benefit packages for all citizens. With a distinctive combination of universalism in financing and marketization in the provision,^26^ Turkey incentivized private investment in the healthcare sector. In addition to public healthcare provision with flat‐rate copayments, the Social Security Institution (SSI) purchases healthcare services from private providers with floating copayments for hospital visits. Hence, the Turkish healthcare system has a competitive internal market that includes both public and private providers.^27^

Turkey reported the first COVID‐19 case in the country on 11 March 2020, later than most European countries. The relatively late arrival of the severe acute respiratory syndrome coronavirus 2 (SARS‐CoV‐2) virus to Turkey provided the country with an opportunity to learn from other countries' experiences with preventive measures. Hence, immediately after the detection of the first case, Turkey adopted a pandemic‐oriented approach to transforming the country's healthcare system. Several measures were introduced to prevent the spread of the virus such as isolations and quarantines if needed, country‐wide contact tracing and routine follow‐ups of all contacted patients by their GPs. During this period, Turkey's relative advantage in addressing the pandemic was the high number of intensive care beds (46 ICU beds per 100,000 individuals)^28^ compared to other OECD countries.^29^ The large‐scale ‘city hospitals’ established with a public–private partnership model have been promoted by the government as the strength of Turkey's healthcare systems since all rooms in the city hospitals could be converted into ICUs.^30^ To make the best of this leverage, the Ministry of Health (MoH) issued a circular on 20 March 2020, stating that ‘all hospitals with at least two specialists from infectious diseases and clinical microbiology, pulmonary medicine or internal medicine, and level 3 intensive care beds qualify as pandemic hospitals’.^31^ Accordingly, all public and private hospitals meeting these conditions started to treat COVID‐19 patients. Additionally, all elective surgeries were cancelled as recommended by the MoH. To prevent the overload on physicians, repeat prescription reports were extended by the SSI and patients were able to receive their medications from pharmacies without seeing their doctors if a consultation was not necessary.

Despite the relatively high number of ICU beds in Turkey, Turkey's healthcare system is characterised by the relative scarcity of medical staff compared to other OECD countries, with 1.9 physicians and 2.3 nurses per 1000 individuals.^32^ The scarcity of medical staff combined with the increasing workload during the pandemic raised concerns about the well‐being of the medical staff and has been a point of weakness in Turkey's response to the COVID‐19 pandemic.^33^

The pandemic‐oriented healthcare services approach raised concerns among doctors about the health conditions of chronic patients in Turkey. The Turkish Medical Association emphasised the risk of increased morbidity for chronic patients caused by delayed diagnosis and treatment.^34^ Calling it a ‘cancer pandemic’, physicians pointed out the risk of an increasing number of late‐diagnosed cancer patients.^35^ They underscored the importance of early diagnosis and routine treatment procedures for better health outcomes.^35^, ^36^ These concerns raised by doctors lead to questions about patient experiences and their access to healthcare services during the pandemic. Patient experiences are multifaceted, which are shaped by disease‐specific conditions and individual circumstances and exacerbated in the cases of complex health situations. To understand patient access and identify the problems in healthcare systems, exploration of patient experiences with their narratives is essential.

This study explores patient experiences during the COVID‐19 pandemic in Turkey, with a focus on patients with chronic or rare diseases considering their complex healthcare needs demanding specialist services. The findings derive from data collected through nine semi‐structured interviews conducted with 10 participants from POs based in Istanbul. Drawing upon studies of the pandemic's impact on patients with chronic or rare diseases, this article aims to contribute to the literature discussing Turkey's healthcare system capacity to meet the needs of citizens with complex healthcare situations as a country that provides universal health coverage.

## 4 METHODS

This study uses qualitative methods to explore patients' experiences of access to healthcare during the COVID‐19 pandemic, as narrated by the members of the POs based in Istanbul, Turkey. In‐depth semi‐structured interviews were conducted over Zoom with 10 respondents from 9 POs based in Istanbul between the period November and December 2020. The interviews were conducted in Turkish and lasted an average of 30 min. They were audio‐recorded with the participants' verbal consent and transcribed verbatim. Ethical approval for this study was granted by the Institutional Review Board for Research in Social Sciences and Humanities of Bogazici University (No: 2020‐54).

The author had a prior relationship with all POs before this study in the context of another research project, but not with all respondents. The POs included in this study involve patients and their relatives in its administration. Among the contacts that the author has, the most active POs that have strong relationships with patients were selected to demonstrate the diverse experiences of patients with different chronic diseases. Thus, the purposive sampling method was used to gather enriched data. The author selected 12 POs to include in this study and sent e‐mails to the official e‐mail addresses of the POs explaining the content of the study and the patient information sheet attached. Among 12 POs, 2 POs did not respond to the e‐mails and 1 PO declined to participate. The remaining nine POs agreed to participate. Table 1 presents the characteristics of the participating POs and respondents.

**Table 1:** Characteristics of POs and respondents

ID	PO focus	Disease type	Respondent characteristic
1	Rare	Metabolic	Patient relative
2	Rare	Metabolic	Patient
3	Chronic	Infectious	Neither
4	Rare	Metabolic	Patient
5	Rare	Muscular	Patient
6	Chronic	Infectious	Patient relative
7	Chronic	Metabolic	Patient
8A & 8B	Rare	Neurologic	Patient & neither
9	Chronic	Metabolic	Neither

Abbreviation: PO, patient organisation.

The interview transcripts were analysed in Turkish using NVivo 12. The author conducted a thematic analysis to code the data following the process described by Braun and Clarke,^37^ using an inductive approach, since the process was driven by data. The author familiarised herself with the interview transcripts, identified ‘pattern responses’^37^ and created codes for overarching themes and subthemes. Data included in this article were translated from Turkish into English by the author.

The findings of this study are subject to limitations. First, the experiences narrated by the participants reflect unique, disease‐specific health issues. Second, the participants were selected from POs based in Istanbul; hence, the experiences might change in different cities of Turkey. Lastly, the data may be subjected to selection bias as the activeness of the POs and their strong network with the patients were selection criteria of participants. However, this is also the strength of the study, since it enables the gathering of enriched information about the diverse issues that patients with chronic or rare diseases face during the pandemic.

## 5 RESULTS

The analysis of the interviews yielded four main themes: Problems in access to healthcare services; lack of clinical information, uncertainty and fear; facilitated access to prescribed medicine; and ongoing informal communication with doctors.

### 5.1 Problems in access to healthcare services

Almost all participants (9) mentioned problems in access to routine and emergency health services caused by the prioritisation of COVID‐19 patients. Two subthemes emerged from the analysis of the interviews: Overcrowding of hospitals with COVID‐19 patients and cancelled or postponed diagnosis and treatment.

#### 5.1.1 Overcrowding of hospitals with COVID‐19 patients

The circular issued by the MoH on 20 March 2020 assigned some public and private hospitals as ‘pandemic hospitals’.^31^ Since patients with chronic and rare disease need speciality services mostly provided by these hospitals, almost all representatives (9) from POs were worried about the inability to find isolated hospitals. These obstacles were aggravated when patients needed to consult some specialities, such as infectious diseases and pulmonary medicine consultants who accept COVID‐19 patients, as stated by a respondent:

Access to infection physicians was quite difficult at the beginning of the pandemic period. Since COVID‐19 is an infection that is covered by the infection unit. Those living with HIV are also treated in the infection unit. So, access to infection physicians was difficult at the beginning of the pandemic period.

(3, chronic disease, infectious, neither patient nor a patient relative).

As the quote above shows, some patients with chronic diseases were not able to consult their doctors, since the physicians were accepting patients with COVID‐19. In some cases, they were not able to get an appointment for vital health problems because of the high number of patients with COVID‐19 at hospitals:

We have a group of patients whose respiratory muscles are paralyzed because of the ALS disease; these patients need to get a ventilator as soon as possible. So, they must continue to live with respiration support. There are two types of it. Either they will have surgery, a hole will be created in their throat as you see on me, or they can get respiration support with a mask before this surgery. To get this, our patients need to have a sleep test at night. Our patients could not get an appointment for this test due to the density of the pandemic.

(8A, rare disease, neurologic, patient).

Despite the widespread concern about the lack of hospitals isolated from COVID‐19, some patients found services more accessible due to their age group:

Because the majority of our group, especially the MPS group, are paediatric patients. In hospitals, as you know, paediatrics departments are cleaner than others, so we can say that they are luckier about that.

(1, rare disease, metabolic, patient relative).

This respondent shares the experience on some patients' inability to access health services when the specialities they have to consult have fewer COVID‐19 patients. The above quote does not imply that paediatrics departments were risk‐free in terms of contacting COVID‐19, but instead that these patients' relatives felt safer about the risk concerning their children, than other patients and their relatives. Additionally, some physicians and hospitals took measures to provide services to their patients in an isolated environment. However, these measures were not enough to relieve the concerns of patients with chronic or rare diseases about their safety from COVID‐19:

Usually, they tried to isolate the oncology department, I mean, I can't say any negative thing about the hospitals on that, they tried to make a separate entrance. But no matter what, the doctors are constantly in touch with other patients at hospitals.

(9, chronic, cancer, neither patient nor a patient relative).

Since COVID‐19 is a communicable disease, some patients were still worried about their health despite isolated departments at hospitals. The concern shared by Participant 9 is legitimate considering the vulnerable health status of cancer patients under treatment.

#### 5.1.2 Cancelled or postponed diagnosis and treatment

Overcrowding of hospitals with COVID‐19 patients resulted in cancellation or postponement of some diagnosis and treatment procedures as stated by most of the respondents:

It prevented early diagnosis. There were serious problems ranging from the disruption of some ongoing treatments to not taking or cutting some medications.

(5, rare disease, muscular, patient).

The diagnosis of rare diseases is a very difficult, time‐consuming process for patients. It requires consultations with different physicians and several medical tests and procedures. Early diagnosis is important for every illness. Delayed diagnosis undermines successful treatment procedures, reduces the quality of life and might decrease life expectancy. The following quote emphasises the importance of early diagnosis:

Early diagnosis of muscle diseases is very valuable. The earlier the patient can be diagnosed, the sooner the patient has the chance to start treatment and the better chance of living a quality life. […] The diagnosis process of many patients was disrupted.

(5, rare disease, muscular, patient).

Respondents reported that some physicians cancelled or postponed appointments with their patients if they worked at hospitals with high numbers of patients with COVID‐19. In these cases, patients felt that physicians had made calculations of the costs and benefits in favour of COVID‐oriented services, and away from chronic patients, as stated by a respondent:

Some of the doctors postponed the treatments if COVID‐19 cases were many in the hospital where they *[patients]* went. They *[doctors]* postponed those which are not urgent. This postponement has negative effects on treatment. After all, it does not show the same effect with the treatment taken in time, but of course… The doctors decided against it as benefit and harm.

(9, chronic, cancer, neither patient nor a patient relative).

However, in some cases, there were barriers to access because of cancelled treatments and surgeries, and here, patients could not access the essential treatment procedures as stated by two respondents from two different patient groups:

There were difficulties in accessing physiotherapy since physiotherapy and rehabilitation centres were closed for a long time, their *[the patients']* physiotherapy was disrupted.

(5, rare disease, muscular, patient).

There are supervised injection services for spinal muscular atrophy (SMA) patients. They could not reach them as they turned into pandemic hospitals; they did not have the chance to obtain the medication in those centres.

(5, rare disease, muscular, patient).

The operations of our patients, whose colostomy bags were opened and whose intestines had to be taken back in, were postponed because it was not urgent.

(8A, rare disease, neurologic, patient).

As the above quotes demonstrate, some patients did not have the chance to access the essential treatments and surgeries because of cancelled treatment and surgeries. For instance, physiotherapy services play a role for patients with muscular diseases in reducing the progression of the disease and improving their health. Inability to access these services can reduce the well‐being of the patients and has the potential to threaten their health status.^5^, ^12^

### 5.2 Lack of clinical information, uncertainty and fear

The beginning of the epidemic was characterised by the lack of accurate clinical information and uncertainty about the pandemic, access to healthcare services and health risks introduced with infection by the SARS‐CoV‐2 virus, which resulted in anxiety and fear among patients about their health. All participants expressed fear caused by the lack of information and the risk of contact with the virus. The uncertainty about the pandemic and the risk of contamination disrupted treatment procedures for some patients:

Most of the patients could not go *[to the hospitals]* because they were afraid and anxious because of the uncertainty, especially in the first period. So, the treatment of our patient group was seriously disrupted, especially in the first half of the pandemic.

(1, rare disease, metabolic, patient relative).

Uncertainty and fear around the patients did not only disrupt their treatment but also aggravated their health problems because of the increasing anxiety. A respondent who is also a patient stated that attacks caused by their disease had become more frequent during the pandemic:

Most of the patients had more attacks because of this uncertainty, their situation at home and their stress.

(4, rare disease, metabolic, patient).

To illustrate the seriousness of the situation, the participant gave an example of their attacks:

My attacks became more frequent. For example, I am having two attacks a week or every week. Normally, I used to have my attacks every six months, every four or five months.

(4, rare disease, metabolic, patient).

The patient experiences narrated by this participant showed that even with their worsening health, the patients were not able to receive immediate treatment because of the fear of going to a hospital and getting infected with COVID‐19.

Participants in the research said that increasing the availability of information and new scientific research on COVID‐19 and specific patient groups had contributed to reducing uncertainties, resulting in the alleviation of anxiety and fear:

The scientific studies have also relieved those living with HIV a little bit because these scientific studies say, HIV+ people with sufficient CD4 have the same risk of being infected with COVID‐19 compared to HIV−. So, what does a person with a sufficient level of CD4 mean? If the person is diagnosed with HIV and receives proper treatment, the CD4 count – the immune cell count – is sufficient, this person has at the same risk as people without HIV. These studies relieved our patients.

(3, chronic disease, infectious, neither patient nor a patient relative).

Narrating the experiences of patients with HIV, the above quote illustrates the potential of reliable scientific information to reduce the widespread anxiety and fear among patients.

### 5.3 Facilitated access to prescribed medicine

To reduce the workload of physicians and shift the human resources to pandemic‐oriented care, the SSI extended the period of repeat prescription reports, which enabled patients to receive their regular medications from pharmacies without seeing their doctors if it was not necessary. For all patient groups who participated in this study, this was seen as a positive development, since it reduced their risk of contracting COVID‐19:

This is a valuable thing indeed. It was really a good thing to extend the report for up to six months, and the patients having access to their medicines without going to the doctor to prescribe their medicines.

(5, rare disease, muscular, patient).

Despite the consensus in the sample about the benefits of facilitated access to prescribed medications, some respondents suggested that it is not a viable option, since medication intakes must be monitored for the health outcomes of the patients:

Now there is no need for a prescription, patients can go to the pharmacy and buy the medicines directly as stated in the circular. But, as I have just said, they do not go to the physician just to get medication. How did that drug affect the body, how many viruses are there in the body, how are the blood values, other additional drugs the patient takes…? Because why are they *[the doctors]* visited every three months or every six months? The drug may not be working at all. The doctor is constantly observing the patient, they can change the medication. Some of our patients use only one pill a day, some of our patients use two or three pills in certain combinations. Frankly, that's why we don't think it is a very sustainable thing to have a prescription without seeing a doctor. Eventually, they should have these tests and examinations done more healthily.

(3, chronic disease, infectious, neither patient nor a patient relative).

As has been identified elsewhere,^12^ this respondent argues that the treatment process of chronic and rare diseases requires routine consultations and medical tests to ensure that the treatment is going well. While the extension of repeated prescription reports reduces the number of hospital visits, patients identified a need to see their doctors in some cases.

### 5.4 Ongoing informal communication with doctors

Patients with chronic or rare diseases in Turkey had close relationships with their doctors due to the long‐term communication that their health condition requires. During the interviews, respondents repeatedly talked about how the doctors communicated with the patients, especially at the beginning of the pandemic:

During this pandemic process those *[the doctors]* who advised us, especially professors from the medical school, frequently held Zoom meetings or live broadcasts from Instagram. The professors gathered, some of them from the paediatrics department, some from others… We all tried to get together at noon or in the evening, at a common time and ask questions quickly.

(4, rare disease, metabolic, patient).

The above quote shows that the virtual meetings arranged by physicians helped patients to obtain information about the pandemic and disease‐specific issues. Those meetings were especially important considering the anxiety and fear caused by a lack of information. However, the information provided by the doctors was not enough at the beginning of the pandemic, since the doctors were also facing uncertainty:

Researcher: Do you think that the information provided by the doctors was helpful to overcome the uncertainty during the pandemic?

Participant: Of course it wasn't since they were also in this uncertainty. So, there wasn't a clear picture neither for the patients nor the doctors, but they did their best to take action not to harm their patients.

(9, chronic, cancer, neither patient nor a patient relative).

The above quote shows that physicians also faced difficulties in providing accurate information to their patients. However, under the guidance of their medical expertise, they provided the best available information to their patients to reduce their uncertainties and fear.

## 6 DISCUSSION

The restructuring of healthcare systems to address the COVID‐19 pandemic has affected the delivery of clinical services to patients who do not have COVID‐19. The findings of this study are in line with the literature on the obstacles caused by the pandemic for patients with chronic or rare diseases. This study provides a patient perspective that underscores that access problems experienced by the patients during the pandemic are a complex mix of factors including shortages and physical barriers, but also perceptions of barriers. Patients' individual experiences with the pandemic, their health situation and perceived contamination risk also shaped their access to healthcare services.

The results suggest that the regulations introduced by the MoH to address the challenges caused by the pandemic created problems in access to routine and emergency health services. Patients who needed to consult some specialities such as infectious diseases and pulmonary medicine consultants who accept COVID‐19 patients were not able to see their doctors. The cancelled or postponed diagnosis procedures involve the risk of worsening the health status of the patients. For instance, considering the rapid deterioration of patients with SMA without appropriate treatment, the inability to access needed care might become life‐threatening. The lack of information and uncertainty at the beginning of the pandemic resulted in anxiety and fear among the patients, which was reduced with the availability of accurate, scientific information in later months. However, even when the services were available, patients were sceptical about face‐to‐face consultations because of the fear of contamination. Some hospitals and physicians took measures to isolate some departments from COVID‐19 patients. However, these measures were not enough, since COVID‐19 is a communicable disease, causing the fear of contact. The extension of repeat prescriptions by the SSI was considered a very positive development that made the lives of patients with chronic and rare diseases easier, reducing their risk of contact with the virus. However, this regulation is not considered a viable long‐term solution since the treatment procedure must be regularly monitored.

The article raises questions about Turkey's ability to provide access to healthcare services for all citizens as a country with UHC. The focus on and prioritisation of the needs of patients affected by the pandemic caused disruptions in the continuum of care for some patients with chronic or rare diseases as the findings of this study demonstrate. The Minister of Health stated that Turkey has managed the pandemic successfully with its robust healthcare system,^30^ with the transformation of all high‐capacity hospitals into pandemic hospitals. However, the findings of this study identify access problems that have been shaped by the multidimensional nature of the healthcare decision‐making process. The Turkish case shows that physicians took initiatives to cancel or postpone appointments for high‐risk chronic patients until they could ensure safer healthcare provision in cooperation with hospitals they work at. While these decisions were in line with WHO guidance,^7^ they were not planned by the MoH and not supported by other mechanisms such as better information provision, introduction of telehealth or online services and creation of self‐management and monitoring plans. The lack of these support mechanisms resulted in uncertainty for patients, causing anxiety about their healthcare situations, and some patients refrained from going to the hospitals even on an urgent basis. Therefore, the findings of this study suggest that the decision‐making process for cancellations or postponements was multi‐layered, shaped by physicians' initiatives and patients' individual experiences with the pandemic. Access problems experienced by patients were a complex mix of factors including shortages and physical barriers, but also perceptions of barriers.

POs can provide insights on disease‐specific experiences and problems that are very valuable to improve access to healthcare services to achieve the UHC target. Considering the access problems for patients with chronic and rare diseases at the beginning of the pandemic, POs could have contributed to the decision‐making process with their knowledge of disease‐specific patient needs. Hence, this article suggests that decision‐making authorities should consult POs to gather information on different needs of patient groups in times of health crises.

This article suggests that the lack of structural policies addressing all dimensions of healthcare systems to ensure access to care for all citizens characterised the pandemic experience for patients who did not have COVID‐19. The complex challenges introduced by the pandemic in Turkey's healthcare system and its pandemic‐oriented restructuring interrupted citizens' healthcare rights. Considering the coexistence of different needs and priorities within the same healthcare system, the findings of this study lead to the question of whether it is possible to ensure every citizen's access to healthcare services during an acute pandemic response. Further research must be conducted to explore this question to address these multidimensional problems caused by the COVID‐19 pandemic and develop policy alternatives for future health system challenges. This article concludes by underscoring the potential contribution of POs to healthcare systems during health crises with their expertise on patient experiences.

## CONFLICT OF INTEREST

The author declares that there is no conflict of interest.

## Data Availability

The data are not publicly available due to privacy and ethical restrictions.
